# Periodontal conditions, oral *Candida albicans *and salivary proteins in type 2 diabetic subjects with emphasis on gender

**DOI:** 10.1186/1472-6831-9-12

**Published:** 2009-05-12

**Authors:** Fawad Javed, Lena Klingspor, Ulf Sundin, Mohammad Altamash, Björn Klinge, Per-Erik Engström

**Affiliations:** 1 Department of Dental Medicine, Division of Periodontology, Karolinska Institutet, Huddinge, Sweden; 2 Department of Laboratory Medicine, Division of Clinical Microbiology, Karolinska University Hospital at Huddinge, Stockholm, Sweden; 3 Department of Laboratory Medicine, Division of Clinical Immunology and Transfusion Medicine, Karolinska University Hospital at Huddinge, Stockholm, Sweden; 4 Altamash Institute of Dental Medicine, Karachi, Pakistan

## Abstract

**Background:**

The association between periodontal conditions, oral yeast colonisation and salivary proteins in subjects with type 2 diabetes (T2D) is not yet documented. The present study aimed to assess the relationship between these variables in type 2 diabetic subjects with reference to gender.

**Methods:**

Fifty-eight type 2 diabetic subjects (23 males and 35 females) with random blood glucose level ≥ 11.1 mmol/L were investigated. Periodontal conditions (plaque index [PI], bleeding on probing [BOP], probing pocket depth [PD] (4 to 6 mm and ≥ 6 mm), oral yeasts, salivary immunoglobulin (Ig) A, IgG and total protein concentrations, and number of present teeth were determined.

**Results:**

Periodontal conditions (PI [*p *< 0.00001], BOP [*p *< 0.01] and PD of 4 to 6 mm [*p *< 0.001], salivary IgG (μg)/mg protein (*p *< 0.001) and salivary total protein concentrations (*p *< 0.05) were higher in type 2 diabetic females with *Candida albicans *(*C. albicans*) colonisation compared to males in the same group. Type 2 diabetic females with *C. albicans *colonisation had more teeth compared to males in the same group (*p *< 0.0001).

**Conclusion:**

Clinical and salivary parameters of periodontal inflammation (BOP and IgG (μg)/mg protein) were higher in type 2 diabetic females with oral *C. albicans *colonisation compared to males in the same group. Further studies are warranted to evaluate the association of gender with these variables in subjects with T2D.

## Background

There is a positive relationship between periodontal inflammation and type 2 diabetes (T2D) [1]. Periodontal inflammation has been shown to be higher in diabetic individuals with random blood glucose level (RBGL) ≥ 11.1 mmol/L compared to individuals with RBGL < 11.1 mmol/L [1]. However, it has been shown that there is no difference in the periodontal status between diabetic subjects with RBGL) ≥ 11.1 mmol/L and non-diabetic individuals [1]. Oral *Candida albicans *(*C. albicans*) colonisation is markedly increased in diabetic compared with non-diabetic individuals and hyperglycemia seems to play a significant role in this regard [2,3]. Diabetes is a metabolic disease characterized by hyperglycemia due to defects in insulin production, insulin action, or both. Diabetes mellitus can impair the function of polymorphonuclear leukocytes which may predispose diabetic patients to greater risk of diseases including periodontal disease and oral candidal infections [2]. There is an indistinct role of oral yeasts in the etiology and pathogenesis of periodontal inflammation, however; *C. albicans *has been isolated from the oral cavities of individuals with severe periodontal inflammation [4]. It has been reported that oral *Candida *colonisation is significantly higher in females compared to males; however, this relationship remains debatable [5,6]. Recent studies have shown that *C. albicans *can colonize the periodontal pockets and is significantly associated with oral mucosal inflammation in females [4,7].

Saliva plays a significant role in maintaining a healthy oral environment. Approximately 95% of the salivary immunoglobulin (Ig) A originates from the salivary gland immunocytes, whereas, most IgG enters the oral cavity by diffusing through the gingival crevices [8]. The concentration of IgG in saliva is normally low, approximately 20 mg/L; however, it is significantly increased in subjects with periodontal inflammation [9,10]. Therefore, a raised IgG concentration in saliva is expected to reflect periodontal inflammation [10,11]. Increased levels of salivary IgA and IgG have been reported in individuals with T2D [12].

Since there is an unclear association between periodontal inflammation, oral yeast colonisation, T2D and gender, the present study aimed to investigate the periodontal conditions, oral yeasts colonization and salivary protein profile in subjects with T2D with emphasis on gender.

## Methods

The study was approved by the regional ethical review board in Stockholm, Sweden and ethical committee of Altamash Institute of Dental Medicine, Karachi, Pakistan. Written information (consent form), printed in simple English and Urdu (native language of Pakistan) was provided. Consenting individuals were invited to an oral healthcare centre for a periodontal examination, collection of oral yeast and unstimulated whole saliva (UWS) samples and measurement of RBGL.

### Inclusion and exclusion criteria

Residents of the Punjab Colony, Karachi, Pakistan with age ranging between 45 to 64 years were included in the study [1,13]. Individuals with medically diagnosed T2D and with a RBGL ≥ 11.1 mmol/L were included. It was mandatory for the participants to have read/understood and signed the consent form before being included in the study.

"Smokers" were defined as individuals smoking at least one cigarette daily for at least six months. Since smoking and use of antibiotics, non-steroidal anti-inflammatory drugs and steroids influence inflammation as well as oral candidal colonisation, the individuals who admitted these behaviours were excluded from the study [2,14,15].

### Study population

A questionnaire survey was conducted in the Punjab colony in which one thousand individuals were interviewed [1,13]. Subjects who reported to have diabetes were requested to present their medical records and/or prescriptions, which confirmed their diabetes status. Among the 1000 adults interviewed, 83 subjects reported to have diabetes, out of which 79 subjects had medically diagnosed T2D. These 79 individuals were invited to an oral healthcare centre for measurement of RBGL, collection of oral yeast and saliva samples and evaluation of periodontal status.

Fifty-eight consenting individuals (23 males and 35 females) satisfied the inclusion criteria and were admitted to the study. There were no significant differences in age, race, ethnicity, socioeconomic variables and living standards among the study population.

### Measurement of random blood glucose levels

Individuals were instructed not to eat or drink at least two hours before their RBGL was recorded. A glucometer (ACCU CHEK, Advantage system/Sensor comfort strips, Roche Diagnostics, Mannheim, Germany) was used to record the RBGL which is a practical method to monitor glycemic levels in individuals with pre-diagnosed diabetes [16-19].

### Collection of oral yeast samples

Participants were instructed to refrain from eating and drinking at least two hours before collection of yeast samples. Sampling was performed between 10:00 am and 1:00 pm.

Each yeast sample was collected by scraping the dorsum of the tongue with a sterile cotton swab (COPAN, Amies Charcoal single swab, CE 0124, Italy). The swabs were returned to the containment tube immediately after sampling. Oral yeasts are pre-dominant on the dorsal surface of tongue; therefore tongue surface scraping is a reliable method for detecting *Candida *species [15].

### Identification of oral yeast samples

Identification to species level was determined by a yeast identification system (API 32-C System bioMériux yeast identification programme, Lyon, France). If identification was not possible with the API 32 system, the yeast isolate was subjected to molecular identification.

For DNA isolation, yeast cells were suspended in 200 μl sterile Polymerase Chain Reaction (PCR)-grade water and genomic DNA was prepared using MagNA pure (Roche Diagnostics GmbH, Mannheim, Germany) a DNA preparation robot [20]. For DNA sequencing and PCR analysis, a region (about 500-bp) of 18S ribosomal ribonucleic acid gene was amplified by PCR using universal primers and ampliTaq Gold DNA polymarase. Primers and free nucleotides from the PCR products were then removed by using QIAquick PCR purification kit (250) (Qiagen, GmbH, Hilden, Germany). The purified PCR products were processed for DNA sequencing by BigDye Terminator Cycle Sequencing using capillary electrophoresis technology in ABI 310 Genetic Analyzer (Applied Biosystems, Foster City, CA). Both strands of PCR amplified DNA fragments were sequenced to avoid error of sequencing [21]. The DNA sequence was analysed by a software and searched in the Blast DNA database for yeast identification and typing [22,23].

### Collection of unstimulated whole saliva samples

The UWS samples were obtained immediately after collection of oral yeasts. To collect the UWS samples, the participants were seated in a bent forward position on a comfortable chair and instructed to spit for five continuous minutes (without swallowing) into a clean plastic funnel connected to a measuring cylinder [24,25]. The volume of saliva was immediately measured and the samples were frozen in disposable 3.5 ml plastic tubes with lid (Sarstedt, Lot: 4071801, Germany). All frozen samples were sealed in an insulated box containing dry ice and transferred to Karolinska University Hospital (Division of Clinical Immunology) Huddinge, Sweden.

### Determination of salivary IgG, IgA and total protein concentrations

Levels of salivary IgG, IgA and total protein concentration were determined as described earlier [11]. In brief, microtiter plates (Corning Inc. NY, USA) were coated with 100 μl per well of anti-human IgG and anti-human IgA (DAKO A/S, Denmark) in coating buffer (0.05 M carbonate-bicarbonate buffer, pH 9.6) and incubated at room temperature for 24 hours. After washing, 100 μL/well of appropriately diluted IgG (Human serum protein calibrator. DAKO A/S, Denmark) and IgA (human colostrum) standards, positive control (saliva from a healthy subject), negative control (saliva from IgA deficient adult subject) and saliva samples were added to the respective microplate wells. After incubation at room temperature, the microplates were washed to remove unbound proteins. Purified alkaline phosphatase conjugated anti-human IgG and IgA (DAKO A/S IgA/AP, Denmark) were added (100 μL/well), and the microplates were incubated for three hours at room temperature. After washing, 100 μL/well of substrate (*p*-nitrophenyl phosphate) in 1.0 M diethanolamine, 0.5 mM MgCl_2_, pH 9.8, (Sigma S-0942) was added. The absorbance was read at 405 nm in a microtiter plate photometer (Molecular devices, Vmax, Sunnyvale, CA, U.S.A).

The bicinchoninic acid, (BCA™) Protein Assay Reagent Kit (Product No. 23227, Pierce Chemical, Co., Rockford, IL, USA), was used to determine the total protein concentration in the saliva supernatants. Using albumin as standard, aliquots of saliva (200 μL/well) were placed in microtiter plates. The protein assay reagent was added, and the plates were incubated at 37°C for 30 minutes. Optical densities were read at 550 nm in a microtiter plate photometer (Molecular devices, Vmax, Sunnyvale, CA, U.S.A).

### Periodontal examination, number of teeth and denture-wearing

A full mouth plaque index (PI), bleeding on probing (BOP) and probing pocket depth (PD) (4 to 6 mm and ≥ 6 mm) were measured at four sites (mesial, distal, buccal and palatal/lingual) of each tooth [1,26-28]. Individuals were investigated for the number of present teeth (excluding maxillary and mandibular third molars) and use of dentures. Teeth with only embedded root remnants were considered as missing. A percentage of the numbers of present teeth was also calculated by the following formula:



### Statistical analysis

The statistical analysis was performed using STATISTICA v. 6.0, (Statsoft, Inc. 1984–2005, Tulsa, OK, USA). The significance of differences of the dependent variables (Ig levels, protein concentration, oral *Candida *colonisation, number of teeth and periodontal conditions) in type 2 diabetic individuals was determined using multiple logistic regression. The independent variables were categorised as dichotomous variables; for example, type 2 diabetic males with *C. albicans *colonisation; 0 and type 2 diabetic females with *C. albicans *colonisation; 1. For multiple comparisons, Bonferroni *Post Hoc *test was performed.

## Results

### Study population

Out of the 58 participants, there were 29 subjects (17 males and 12 females) with *C. albicans *colonisation. In this group, the mean age of males and females was 50.5 years (range 45–64 years) and 49.3 years (range 45–59 years) respectively. In subjects without *C. albicans *colonisation (n = 29), there were six males and 23 females. The mean ages of males and females in this group were 50.6 years (range 45–56 years) and 51.1 years (range 47–59 years) correspondingly.

The durations of T2D in individuals with and without *C. albicans *colonisation were 10.5 (range 8–14 years) and 10.8 years (range 8–12 years) correspondingly.

### Oral yeast colonisation in subjects with T2D

**Figure 1:**
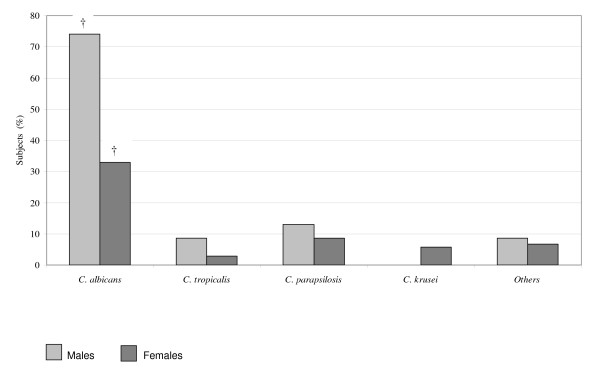
**Oral *Candida (C.) *colonisation in relation to gender among type 2 diabetic subjects**. † *P *< 0.01. Others (males): *C. lusitaniae *Others (females): *C. kefyr*. † Differences in oral *Candida albicans *(*C. albicans*) colonisation between type 2 diabetic males and females were tested using multiple logistic regression. For multiple comparisons, Bonferroni *Post Hoc *test was performed.

Oral *C. albicans *colonisation was significantly higher in type 2 diabetic males compared to females as shown in Figure 1 (*p *< 0.01).

### Prevalence of denture-wearing

In type 2 diabetic subjects with *C. albicans *colonisation, denture-wearing was more frequent in males (47%) compared to females (16.6%). There was no difference in denture-wearing in type 2 diabetic subjects without *C. albicans *colonisation (data not shown).

### Salivary flow rate (SFR), salivary IgG (μg)/mg protein, IgA (μg)/mg protein and total protein concentration in relation to C. albicans colonisation, number of teeth and gender

Type 2 diabetic females had a lower SFR (mean 0.15 ml/min; range 0.1–0.3 ml/min) compared to males with T2D (mean 0.38 ml/min; range 0.2–0.5 ml/min) (*p *< 0.05).

**Figure 2:**
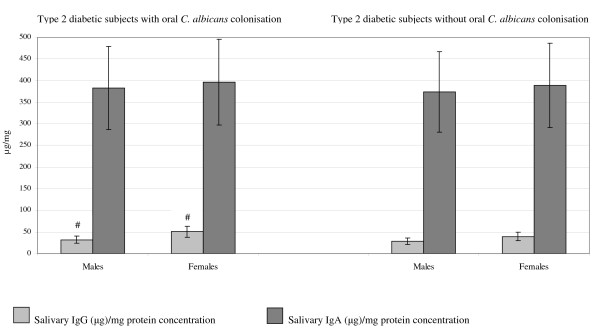
**Salivary IgG (μg)/mg protein and IgA (μg)/mg protein concentrations in type 2 diabetic subjects with and without oral *C. albicans *colonization in relation to gender**. # *p *< 0.001 indicates a higher concentration of salivary IgG (μg)/mg protein concentration in type 2 diabetic females compared to males with T2D and oral *C. albicans *colonization. Differences in levels of salivary IgG (μg)/mg protein and IgA (μg)/mg protein in type 2 diabetic males and females with and without oral *Candida albicans *(*C. albicans*) colonisation were tested using multiple logistic regression. Data are mean ± 2 standard deviations.

**Figure 3:**
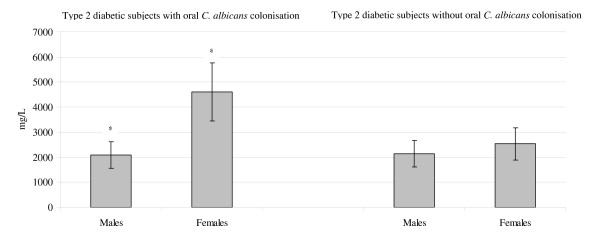
**Salivary total protein concentrations in type 2 diabetic subjects with and without oral *C. albicans *colonisation in relation to gender**. * *P *< 0.05. Differences in salivary total protein concentrations in type 2 diabetic males and females with and without oral *Candida albicans *(*C. albicans*) colonisation were tested using multiple logistic regression. Data are mean ± 2 standard deviations.

Females with *C. albicans *colonisation had higher levels of IgG (μg)/mg protein (*p *< 0.001) and total protein concentration (*p *< 0.05) compared to males with *C. albicans*. These results are shown in Figure 2 and Figure 3. The females also had more teeth (mean number of teeth19.5; range 13–21 teeth) compared with the males (mean number of teeth 12; range 10–16 teeth) (*p *< 0.0001).

Among type 2 diabetic males and females without *C. albicans *colonisation, salivary IgG (μg)/mg protein levels were 32.2 μg/mg (range 7–107.7 μg/mg) and 38.6 μg/mg (range 2.8–79.3 μg/mg) correspondingly. In these subjects, mean salivary IgA (μg)/mg protein levels were 364.9 μg/mg (range 155–1489 μg/mg) and 391.8 μg/mg (range 90–1863 μg/mg). Salivary total protein concentrations, in these individuals, were 2181 mg/L (range 879.5–3812.3 mg/L) and 2569.4 mg/L (range 1914.8–3107.7 mg/L) respectively.

Mean number of teeth in type 2 diabetic males and females without *C. albicans *colonisation were 12.1 (range 9–15) and 15.6 teeth (range 9–16) respectively.

### Periodontal conditions and number of teeth in relation to *C. albicans *colonisation and gender

**Figure 4:**
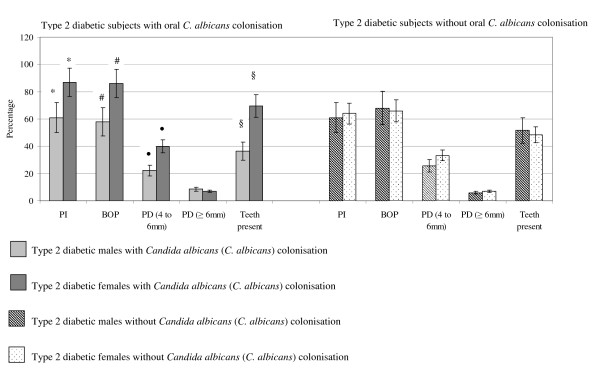
**Periodontal conditions in type 2 diabetic subjects with and without oral *Candida albicans (C. albicans) *colonisation with respect to gender. Data are mean ± 2 standard deviations**. * *p *< 0.00001 # *p *< 0.01 • *p *< 0.001. §*p *< 0.0001. PI: Plaque index (%). BOP: Bleeding on probing (%). PD: Probing pocket depth (%). Differences between PI, BOP, PD (4 to 6 mm and ≥ 6 mm) between type 2 diabetic males (n = 17) and females (n = 12) with *Candida albicans *(*C. albicans*) colonisation were tested using multiple logistic regression. Data are mean ± 2 standard deviations.

Type 2 diabetic females with *C. albicans *colonisation had higher PI (*p *< 0.00001), BOP (*p *< 0.01) and PD (4 to 6 mm) (*p *< 0.001) compared with males in the same group. These females had more teeth (mean number of teeth 19.5; range 16 to 26) compared to type 2 diabetic males with *C. albicans *colonisation (mean number of teeth 12; range 10 to 16) (*p *< 0.0001). These results are shown in Figure 4.

## Discussion

Among non-diabetic individuals, *C. albicans *colonisation has been reported to be higher in dentate females compared to males [6]. The current study showed that clinical and salivary parameters of periodontal inflammation (BOP and IgG per milligram of the salivary total protein concentration [IgG (μg)/mg protein]) are elevated in type 2 diabetic females with oral *C. albicans *colonisation compared to males in the same group.

It is known that diabetic individuals have a reduced SFR compared to non-diabetic controls and is independent of glycemic levels [29,30]. In the present study, males and females had similar casual plasma glucose levels; however, the SFR was almost twice as high in males compared with females. An explanation that has been given in this context is that the size of salivary glands is smaller in females compared with males [31]. A raised salivary IgG concentration has been reported in patients with T2D [12]. It is noteworthy that a diminished SFR concentrates the salivary proteins, thereby expressing raised concentrations of salivary proteins including IgA and IgG. Therefore, salivary IgA and IgG concentrations should be expressed as IgA (μg)/mg protein and IgG (μg)/mg protein, to normalize against volume. An increased level of salivary IgG (μg)/mg protein concentration reflects a raised oral inflammation [11]. The intensity of periodontal inflammation has been associated with the number of teeth affected [32]. This reflects that a greater number of teeth with inflamed periodontal tissues allow an extensive leakage of IgG into the oral cavity through the gingival crevices. In subjects with *C. albicans *colonisation, levels of IgG (μg)/mg protein were almost twice as high in females compared with males. However it is noteworthy that these females had nearly twice as many teeth as males in the same group. Therefore, among type 2 diabetic subjects with *C. albicans *colonisation, the presence of more teeth seems to be the most likely explanation for the higher IgG (μg)/mg protein levels in females compared with males.

Other factors that may influence oral candidal colonisation include denture-wearing, xerostomia and age [33-35]. In the current study, nearly 74% of the males were harbouring oral *C. albicans *compared to 23% in females. This is in accordance with another study where *C. albicans *colonisation was dominant among males (83%) compared to females (56%) [36]. It has been shown that oral *Candida *colonisation can increase up to six-fold in denture-wearers [37]. The current results showed that in subjects with *C. albicans *colonisation, denture wearing was more frequent in males (47%) compared to females (16.6%). An explanation in this context may be that dentures (either partial or complete) obstruct the salivary flow from minor salivary glands and the free exchange of oxygen. Thus the resultant low pH level facilitates the growth of *C. albicans *[37]. In the present study, among subjects with *C. albicans *colonisation, males had a higher SFR compared to females. However, these flow rates were lower than the SFR in non-diabetic individuals (0.5 ml/min) [38]. It is known that there is an inverse relationship between SFR and oral candidal colonisation. However; despite having a higher SFR compared to the females, males had increased *C. albicans *colonisation compared with females. This may once again be associated with denture-wearing which was approximately three times more common in type 2 diabetic males (47%) compared to females (16.6%) with T2D.

There is a positive relationship between *C. albicans *colonisation and age [35]. However; in the current study age can not be the effect on the differences in *C. albicans *colonisation, as it was adjusted in both groups.

The present results showed a higher PI, BOP and PD (4 to 6 mm) in females with *C. albicans *colonisation compared to males. However, it is notable that these females had a lower SFR and had approximately twice as many teeth as males. Therefore, a higher number of teeth and reduced SFR may possibly be associated with increased periodontal conditions and salivary IgG (μg)/mg protein and total protein concentration in these females compared with males.

## Conclusion

Clinical and salivary parameters of periodontal inflammation (BOP and IgG (μg)/mg protein) were higher in type 2 diabetic females with oral *C. albicans *colonisation compared to males in the same group. These gender-specific features may offer a route to improve oral healthcare for females with T2D. However, further studies are warranted in this regard.

## Competing interests

The authors declare that they have no competing interests.

## Authors' contributions

FJ performed the clinical and salivary investigations, carried out the statistical analysis, evaluated results and wrote the manuscript. LK carried out the mycological investigations and contributed in manuscript writing and revision. US contributed to salivary investigations, manuscript writing and revision. MA helped in clinical periodontal evaluation of the study subjects. BK contributed to manuscript writing as well as revision. PE-E supervised this project and contributed to salivary investigations, evaluation of results, manuscript writing and revision. All authors read and approved the final manuscript.

## Pre-publication history

The pre-publication history for this paper can be accessed here:


